# Signed, sealed and delivered: “big tobacco” in Hollywood, 1927–1951

**DOI:** 10.1136/tc.2008.025445

**Published:** 2008-09-18

**Authors:** K L Lum, J R Polansky, R K Jackler, S A Glantz

**Affiliations:** 1 Center for Tobacco Control Research and Education, University of California, San Francisco, California, USA; 2 Onbeyond LLC, Fairfax, California, USA; 3 Department of Otolaryngology – Head & Neck Surgery, Stanford University School of Medicine, Stanford, California, USA; 4 Center for Tobacco Control Research and Education and Department of Medicine, University of California, San Francisco, California, USA

## Abstract

**Objective:**

Smoking in movies is associated with adolescent and young adult smoking initiation. Public health efforts to eliminate smoking from films accessible to youth have been countered by defenders of the status quo, who associate tobacco imagery in “classic” movies with artistry and nostalgia. The present work explores the mutually beneficial commercial collaborations between the tobacco companies and major motion picture studios from the late 1920s through the 1940s.

**Methods:**

Cigarette endorsement contracts with Hollywood stars and movie studios were obtained from internal tobacco industry documents at the University of California, San Francisco (UCSF) Legacy Tobacco Documents Library and the Jackler advertising collection at Stanford.

**Results:**

Cigarette advertising campaigns that included Hollywood endorsements appeared from 1927 to 1951, with major activity in 1931–2 and 1937–8 for American Tobacco Company’s Lucky Strike, and in the late 1940s for Liggett & Myers’ Chesterfield. Endorsement contracts and communication between American Tobacco and movie stars and studios explicitly reveal the cross-promotional value of the campaigns. American Tobacco paid movie stars who endorsed Lucky Strike cigarettes US$218 750 in 1937–8 (equivalent to US$3.2 million in 2008) for their testimonials.

**Conclusions:**

Hollywood endorsements in cigarette advertising afforded motion picture studios nationwide publicity supported by the tobacco industry’s multimillion US dollar advertising budgets. Cross-promotion was the incentive that led to a synergistic relationship between the US tobacco and motion picture industries, whose artefacts, including “classic” films with smoking and glamorous publicity images with cigarettes, continue to perpetuate public tolerance of onscreen smoking. Market-based disincentives within the film industry may be a solution to decouple the historical association between Hollywood films and cigarettes.

Smoking in movies is a major reason for adolescent^1^^–^4 and young adult^5^ smoking initiation. Because there is a dose–response relationship in the effect of smoking in movies on adolescent smoking, public health authorities have urged that smoking be removed from films rated for youth audiences by rating future movies with smoking “R” in the USA (or “18” in the UK or “18A” in Canada). Such a change would reduce adolescent exposure to smoking by about 60% and prevent an estimated 200 000 youth from starting to smoke in the US alone.^6^ ^7^

Paid product placement of tobacco products in movies between 1970^8^ and the mid-1990s^9^ is well documented. Nevertheless, when public health experts call for the film industry to eliminate smoking from future movies accessible to youth,^6^ defenders of the status quo argue that smoking has been prominent on screen since the silent film era^10^ and that tobacco imagery is integral to the artistry of American film, citing “classic” smoking scenes in such films as *Casablanca* (1942) and *Now, Voyager* (1942).^11^^–^13 This argument does not consider the possible effects of commercial relationships between the motion picture and tobacco industries during this period. This paper examines the relationship between the motion picture and tobacco industries during the “studio system” era, when major film companies held actors to multiyear contracts and controlled most first-run movie theatres.

## METHODS

Internal tobacco industry documents at the University of California, San Francisco (UCSF) Legacy Tobacco Documents Library (http://www.legacy.library.ucsf.edu) were obtained through keyword searches, including “movie endorsement”, “agreement”, “testimonial”, “Hollywood” and “screen/movie star” and major studio names (e.g., “Paramount”, “Warner Bros”) between November 2007 and February 2008. The snowball method and surrounding Bates number searches were used to investigate the evolution of certain endorsement contracts or advertising campaigns. Endorsement contracts were also related to additional cigarette advertisements found from a review of the Robert Jackler collection of tobacco advertisements (http://tobacco.stanford.edu) in January and July 2008. Online archives of the *Los Angeles Times* and *New York Times* were searched using such terms as “testimonial”, “endorsement” and “tobacco advertising”. Relevant advertising budgets were obtained from the US Census Bureau’s *Statistical Abstract* and the marketing journal *Printer’s Ink*. In total, 246 archival documents were ultimately analysed. Movie, studio and actor details were obtained from the Internet Movie Database (http://www.imdbpro.com). US dollar values were adjusted for inflation to 2008 equivalents using the average Consumer Price Index for the relevant year.

Keyword searches found that tobacco company print and radio endorsement contracts with motion picture figures, and related studio correspondence were concentrated between 1927–1951; from the advent of “talking” motion pictures to the rise of television. The number of Hollywood endorsements in print adverts and radio broadcasts between 1927–1951 was determined by reviewing cigarette advertisements and radio program transcripts in the Legacy Tobacco Documents Library and the Jackler collection. Endorsements were defined by endorser, advertising copy, movie tie-in and accompanying contract agreement, and counted in the year of first appearance. The extent to which major studios engaged in tobacco cross-promotion was determined by the number of times a specific studio was mentioned in a cigarette print advert or Hollywood guest star appearance on a tobacco-sponsored radio program. Monetary considerations made to Hollywood stars for their testimonials were taken from endorsement agreements and converted into 2008 US dollar equivalents.

## RESULTS

**Figure 1:**
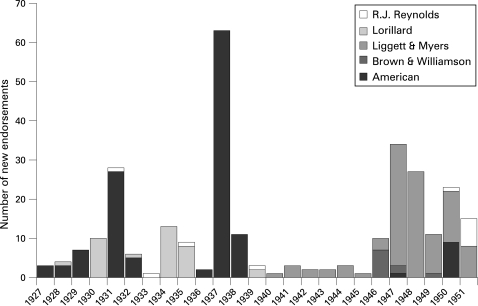
Hollywood endorsements in cigarette advertising were pervasive from 1927–1951. The number of unique endorsements reflects the number of Hollywood testimonials received and used in advertising that could be counted in existing records of print adverts and radio broadcast transcriptions from previously secret tobacco industry documents (total = 292). American Tobacco Company dominated the early period of cross-promotional cigarette advertising for its Lucky Strike brand, most notably in the 1937–8 campaign that focused on the importance of voice to movie actors and directors. When American Tobacco fell under investigation by the Federal Trade Commission for its misleading advertising, Liggett & Myers was free to conduct similar advertising campaigns for its Chesterfield brand during the late 1940s.

Cross-promotion arrangements (then termed “tie-ins”, “tie-ups” or “exploitation”) generating publicity for tobacco companies and studios originated from cigarette advertising featuring testimonials from stage celebrities such as Florenz Ziegfield and Helen Hayes.^11^ Cigarette advertising campaigns exploiting Hollywood celebrity while promoting films from the major studios appeared from 1927 to 1951, but creation of new adverts peaked in three major campaigns: 1931–2 and 1937–8 for American Tobacco’s Lucky Strike and in the late 1940s for Liggett & Myers’ Chesterfield (fig 1).

### Tobacco companies give Hollywood national advertising

Advertising-driven competition among Lucky Strike, Chesterfield and Camel cigarette brands made the tobacco industry among the biggest advertisers in the USA. In 1929, American Tobacco spent US$6.5 million (US$80 million in 2008) on print and radio advertising, more than three times the US$1.9 million (equivalent to US$23 million in 2008) RJ Reynolds spent on Camels, the leading brand. In the worsening Great Depression of 1930, American Tobacco’s Lucky Strike boosted its print and radio advertising budget by 53% (US$126 million in 2008), gaining market share from Camel and Chesterfield to win first place.^14^

By contrast, the motion picture industry relied on modest “co-op” spending (budgeted promotional campaigns with dual benefit to the vendor and retailer) for theatre listings, trailers of coming attractions, lobby posters and word of mouth.^15^ Due to national advertising opportunities afforded by the tobacco industry, major studios maximised exposure for their stars, who “sold” the studios’ pictures to the public, in promotional broadsides timed to the opening dates of their large budget “A” class films.

### American Tobacco exploits “talkies”, 1927

American Tobacco, one of the leading cigarette companies of its day,^16^ was well positioned to out-advertise its competition using innovative multimedia campaigns. Retained by American Tobacco in 1925,^16^ Lord & Thomas advertising agency by 1927 also represented Radio Corporation of America (RCA), the parent corporation of the National Broadcasting Company, and RKO, an RCA subsidiary and one of Hollywood’s major film studios.^17^ Later, Paramount Pictures, another major studio, became a client of Lord & Thomas.^16^ One of the largest advertising agencies, Lord & Thomas ran American Tobacco’s campaigns until its successor entity, Foote Cone & Belding, resigned the account in 1948.

**Figure 2:**
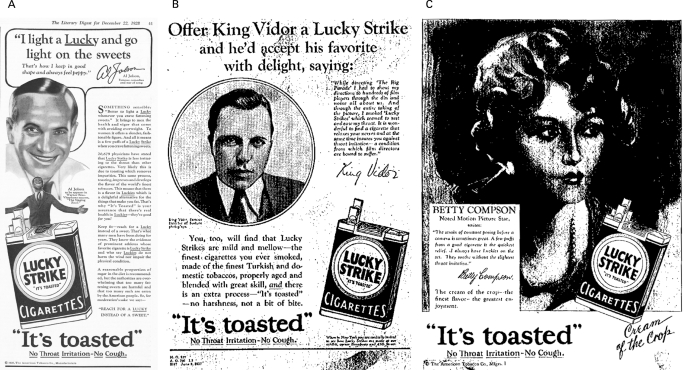
Hollywood movie stars and directors endorse Lucky Strike cigarettes. A. Al Jolson, the famous actor/singer star of the first talking picture, *The Jazz Singer* (1927), appeared in this 1928 advertisement endorsing Lucky Strike as an alternative to fattening sweets. In smaller print, the studio tie-in states, “Al Jolson, as he appears in Warner Bros Vitaphone success, “The Singing Fool [1928]””. This advertisement belonged to the “Reach for a Lucky Instead of a Sweet” campaign in 1928–1929.^18^ ^103^ B. King Vidor, a prominent film director, endorsed Lucky Strike cigarettes for their soothing qualities in this 1927 “Precious Voice” campaign advertisement. Vidor’s testimonial includes tie-in for his movie, *The Big Parade* (1925).^20^ C. Betty Compson, a successful actress who made the crossover to sound, endorsed Lucky Strike (commonly known as “Luckies”) in this 1928 advertisement in the “Cream of the Crop” series. Compson’s testimonial describes the relief she gets from smoking Luckies, which she always has on hand “on the set”.^104^

Following Warner Bros’ 1927 release of *The Jazz Singer*, the world’s first synchronised “talking picture” that made movies into a mass phenomenon, American Tobacco sought Hollywood endorsements for an ongoing campaign that claimed Lucky Strike spared smokers’ throats and protected their voices (table 1). The focus on show business and its personalities differentiated American Tobacco’s celebrity testimonials from other tobacco companies, which weakly copied American Tobacco’s innovation before the 1940s. In a 1928 Lucky Strike advert featuring actor Jimmy Gleason’s testimonial and plugging his Broadway show, Gleason stated, “[Lucky Strike] is certainly the cigarette of the acting profession”.^18^ American Tobacco documents contain dozens of testimonials, authored by Lord & Thomas but signed by famous names in vaudeville and the legitimate theatre, including composer George Gershwin, producer Sam Harris, actress Helen Hayes and *Jazz Singer* star Al Jolson (fig 2A).

**Table 1:** Cigarette advertising campaigns using actor endorsements, 1927–1950

Date	Brand, campaign	Company	Typical advertising copy	
			Headline	Testimonial and/or Hollywood tie-in
Jan–Jun 1927	Lucky Strike, Precious Voice^20^	ATC	“The Captivating Voice of the Delightful Actress, Alice Brady”	“I use Lucky Strikes, as I find they not only protect my voice but afford me the greatest amount of genuine enjoyment”
Jul–Nov 1927	Lucky Strike, Testimonial Series (Double and Group)^21^	ATC	“I got the idea from Florenz Ziegfeld”	“Several years ago, when I first began to smoke Lucky Strikes, I noticed that my voice remained unirritated after a most strenuous time directing rehearsals”
Nov–Dec 1927	Lucky Strike, Testimonial Series (Package)^21^	ATC	“Paul Leni, Motion Picture Director, writes:”	“While directing the filming of “The Cat and the Canary” for Universal Pictures Corporation, I was fortunate to always have a supply of Lucky Strikes on hand”
Jan–Apr 1928	Lucky Strike, Cream of the Crop Series (Testimonial Series)^22^	ATC	“I Always Have Luckies, Says Betty Compson, Motion Picture Star”	“The strain of constant posing before a camera is sometimes great. A few puffs from a good cigarette is the quickest relief. I always have Luckies on the set”
Jun–Aug 1928	Lucky Strike, Cream of the Crop Series (Frames Series)^22^	ATC	“Cream of the Crop”	“I get more kick from the Lucky Strike flavor than from any other cigarette”—Douglas Fairbanks, “America’s Motion Picture Favorite, as he will appear in…“The Iron Mask””
1930	Old Gold, They Gave a New Thrill^23^	LOR	“They gave a *new* Thrill. That’s why they got there….so quickly”	“Joan Crawfords [*sic*] and Old Golds are Nature’s favorites”. “[Joan’s] recent picture, “Our Blushing Brides”, is a nationwide hit”
Sept–Dec 1931	Lucky Strike, Modern Testimonials Series^24^	ATC	“I have to be kind to my throat”	“I’ve tried several brands of cigarette but I prefer Luckies. I smoke them regularly as I have to be kind to my throat”—Kay Francis, “…one of Warner Bros’ brightest stars”
Jan–Feb 1932	Lucky Strike, Frame Series (Movie Stars)^25^,^26^	ATC	“There’s none so good as Luckies”	“Put me down as one who always reaches for a Lucky. It’s a real delight to find a Cellophane wrapper that opens without an ice pick”—Jean Harlow, appearing “…in her new Columbia Picture, “Three Wise Girls…””
1934–1935	Old Gold, The Throat-ease Cigarette	LOR	“Do women smokers realize what Old Gold’s throat-ease means?…”	“…asks Barbara Stanwyck [Old Gold smoker since 1933]…Barbara Stanwyck starring in Warner Bros forthcoming picture, “The Lost Lady””
Jan–Apr 1937	Lucky Strike, Precious Voice^27^	ATC	“Hollywood’s Most Polished Voice”	“…I find that Luckies are always gentle on my throat. It’s only common sense for an actor—or anyone else, for that matter—to want a light smoke”—Herbert Marshall, “co-starring with Barbara Stanwyck in RKO’s “A Love Like That””
Jun–Oct 1937	Lucky Strike, Testimonial Strip^28^,^29^	ATC	“She often acts 12 hours a day! CAROLE LOMBARD tells how her singing teacher urged her to chose a light smoke—Luckies…”	“In making “Swing High, Swing Low”, my recent Paramount picture”, says Carole Lombard, “there was an unusual strain on my throat…I could smoke Luckies all day without the slightest throat irritation. Most others on the set also prefer them”
Jan–Feb 1938	Lucky Strike, Tobacco Expert and Voice^28^	ATC	“Her Throat Insured for $50 000”	“…I take no chances on an irritated throat. No matter how much I use my voice in acting, I always find Luckies gentle”—Dolores Del Rio, “starring in the 20th Century Fox Picture, “Shanghai Deadline””
1940–1950	Chesterfield, various campaigns	L&M	“ABC: Always Buy Chesterfield”	“All my friends know Chesterfield is my brand”—Rita Hayworth, “star of Columbia’s Technicolor Production “Down to Earth””
1946–1947	Raleigh, Less Nicotine/Less Throat Irritants^30^	B&W	“Less Nicotine, Less Throat Irritants”	“I’d rather have a Raleigh!”—Herbert Marshall, “starring in Duel in the Sun, a David O’Selznick Production”
1949–1950	Camel, Camels for mildness^31^	RJR	“How *MILD* can a cigarette be?”	“My throat sure gets a workout, so it’s easy to see why I smoke the mild cigarette...CAMEL!”—Peter Lind Hayes
Feb–Apr 1950	Lucky Strike, Rough Puff^32^^–^37	ATC	“There’s never a rough puff in a Lucky”	“Hedy Lamarr says: “A good cigarette is like a good movie—always enjoyable. That’s why it’s Luckies for me!””
1950	Camel, 30-Day Camel Mildness Test^38^	RJR	“With Stars who must think of their throats, it’s Cool, Mild Camels!”	“John Wayne, Movie Hero: “The roles I play are far from easy on my voice! Camels suit my throat to a “T”!””

Source: American Tobacco Company,^39^^–^41 RJ Reynolds,^38^ Jackler Collection.

“Luckies” is a name often used to refer to the Lucky Strike brand. ATC, American Tobacco Company; B&W, Brown & Williamson; L&M, Liggett & Myers; LOR, Lorillard; RJR, RJ Reynolds.

Since the transition to sound was just beginning, Hollywood film directors were the first film figures to appear in Lucky Strike advertisements (table 2). The major studio employing the director authorised his testimonial, written by the advertising agency, and ensured mention of the title of the director’s motion picture to be promoted in the ad.^19^ Metro–Goldwyn–Mayer (MGM) director King Vidor was featured in a 1927 advert that included his photograph, signature, plug for his silent film hit *The Big Parade*, and testimonial stating: “It is wonderful to find a cigarette that relaxes your nerves and at the same time insures you against throat irritation—a condition from which film directors are bound to suffer”^20^ (fig 2B). The cross-promotion pattern was set in these early adverts.

**Table 2:** Hollywood directors in Lucky Strike adverts, 1927–8

Name	Studio affiliation	Known for:
Herbert Brenon	Paramount	Sorrell and Son (1927)
Allan Dwan	Fox and others	Sands of Iwo Jima (1949)
Paul Leni	Universal	The Cat and the Canary (1927)
Fred Niblo	MGM and others	Ben-Hur (1925)
Albert Parker	United Artists	The Black Pirate (1926)
Edward Sutherland	Paramount and others	Abie’s Irish Rose (1946)
King Vidor	MGM and others	Duel in the Sun (1946)
Raoul Walsh	Various	Sadie Thompson (1928)

Source: American Tobacco,^19^ ^21^ and http://IMDbPro.com.

Lucky Strike’s 1927 campaign also associated attractive qualities of female actors and their voices with smoking Luckies.^20^ Lord & Thomas used “good, wholesome American actresses like Alice Brady” in a campaign that was, according to *Fortune* magazine, “so well timed…that public cigarette smoking by women in America can be correctly dated from [1927]”.^16^ Placed above the headline, “The Captivating Voice of the Delightful Actress, Alice Brady”, Brady’s testimonial read, “I use Lucky Strikes, as I find they not only protect my voice but afford me the greatest amount of genuine enjoyment”.^20^ Stage and screen actress Betty Compson signed a testimonial that read, “The strain of constant posing before a camera is sometimes great…I always have Luckies on the set”^19^ (fig 2C).

### Federal Trade Commission scrutinises cigarette advert testimonials, 1929

Protesting Lucky Strike’s 1928 endorsement adverts bearing the slogan, “Reach for a Lucky instead of a sweet”, the US candy industry lobbied federal regulators to restrict American Tobacco’s use of this phrase.^42^ The Federal Trade Commission concluded that American Tobacco’s advertising was misleading in several respects.^43^ Some Lucky Strike testimonials were from non-smokers, while others were not written or reviewed by the celebrities represented as making them.^44^ The FTC specifically cited the endorsement credited to *Jazz Singer* star Al Jolson:

Talking pictures demand a very clear voice…Toasting kills off all the irritants, so my voice is as clear as a bell in every scene. Folks, let me tell you, the good old flavor of Luckies is as sweet and soothing as the best “Mammy” song ever written…There’s one great thing about the toasted flavor…it surely satisfies the craving for sweets. That’s how I always keep in good shape and always feel peppy.^44^

The FTC found that American Tobacco had authority to use this statement, and paid for it, but that Jolson did not prepare or review it 44 before its use in a 1928 *Lucky Strike Radio Hour* broadcast.^45^ Instead, Warner Bros’ advertising manager A P Waxman^46^ signed a release on Warner Bros letterhead for text similar to what was used on air, stating that he acted on Jolson’s behalf.^18^

In November 1929, the FTC issued a cease and desist order against American Tobacco, prohibiting testimonials unless written by the endorser, whose opinions were “genuine, authorised and unbiased”.^44^ The FTC ordered American Tobacco to conspicuously disclose payments for testimonials in its advertising.^44^ However, American Tobacco successfully removed this disclosure stipulation in 1934.^44^ No tobacco company acknowledged in its print adverts or radio broadcasts that advertising testimonials were bought or that an advertising agency drafted them.^47^

### Lucky Strike revives the Hollywood testimonial, 1931

American Tobacco revised the contractual language for its 1931 endorsement campaign to ensure control over the language and messaging of the testimonials, while still conforming to the FTC’s 1929 stipulations that endorsers supply the testimonial.^48^ Actors signed a revised release that read:

No monetary or other consideration of any kind or character has been paid me or promised me for the above statement, by Mr. [American Tobacco’s agent] or by the manufacturers of Lucky Strike Cigarettes or otherwise.^19^

While actors offered their opinions and declared the number of years they smoked Luckies, they permitted Lord & Thomas to write the actual testimonial, “phrased in such form as to make an effective message from the standpoint of truthfulness and advertising value”.^48^

American Tobacco’s endorsement contracts also specified the use of the stars’ names, photographs and conspicuous mention of film title and studio “in advertisements of LUCKY STRIKE Cigarettes, in newspapers, magazines, on billboards, over the radio and/or in any other media of advertising”.^48^ Before publication, the studios reviewed and approved all advertising copy, including any other names mentioned in connection with the star, studio or motion picture plugged. In 1931, for example, Warner Bros and Paramount publicists sent letters to fan magazine *Photoplay* approving use of co-stars and star’s spouses in specific Lucky Strike adverts.^48^ (*Photoplay* acted as one of American Tobacco’s agents in securing 1929–1931 Hollywood endorsements, reportedly in exchange for an advertising appropriation of the 2008 equivalent of US$694 000.)^48^^–^50 However, to participate in this lucrative partnership, the studios bypassed their own ban on actor endorsements, promulgated in 1931 by the Motion Picture Producers and Distributors of America (MPPDA, precursor to the Motion Picture Association of America (MPAA)).^51^

Following publicity from the 1929 FTC inquiry and the MPPDA’s 1931 rule against paid endorsements by stars, the 1931 Lucky Strike campaign explicitly denied that endorsers were bought. A newspaper advert featuring Mary Astor, a Radio Pictures contract player, asked:

Is Miss Astor’s Statement Paid For? You may be interested in knowing that not one cent was paid to Miss Astor to make the above statement. Miss Astor has been a smoker of Lucky Strike cigarettes for over a year. We hope the publicity herewith given will be as beneficial to her and to Radio Pictures, her producers, as the endorsement of LUCKIES is to you and to us.^24^

This explanation reassured the reader and suggested that American Tobacco made arrangements with the studios that contractually controlled the endorsements from its actors, rather than with actors directly. The statement also spotlights the cross-promotional value of cigarettes to Astor’s studio employer, perhaps to aid American Tobacco in soliciting cooperation from other studios.

### Lucky Strike’s new Hollywood campaign, 1937–8

With the 1934 removal of the FTC’s stipulation that testimonial payments be disclosed, the process of buying testimonials from top stars was discussed openly in 1937 meeting minutes and memoranda from Lord & Thomas’ Lucky Strike Group.^52^^–^57 The advertising agency set the price of the endorsement, then determined the endorser’s smoking status, brand preference and willingness to endorse Lucky Strike. An interview protocol captured the prospect’s answers to key questions, without closing the doors to a testimonial:

1. Does signer smoke Luckies?

2. Does signer smoke Luckies exclusively?

3. If answer to Question 2 is “No:”

(a) does the signer smoke Luckies consistently and other brands occasionally?

(b) will signer give full preference in smoking to Luckies henceforth?^58^

For Hollywood and American Tobacco, the 1937–8 Lucky Strike campaign was based on “mutual using”.^52^ ^59^ ^60^ Each studio aimed to maximise its exposure in national cigarette campaigns, for competitive advantage over other studios. American Tobacco aimed to exploit Hollywood’s top stars, regardless of their studio affiliation. For Lord & Thomas, the interests of the stars and studios were secondary to Lucky Strike’s sales goals. Albert Lasker, president of Lord & Thomas, reminded his Lucky Strike Group in January 1937:

[T]he most important thing about this campaign, gentlemen, is what *we say* in the testimonials. That’s where we do our selling…This is a most serious thing and requires much concentration and thought.^52^

The Lucky Strike Group also tried to balance the studio’s requirement of a “plug” with the agency’s desire to focus the reader on the Lucky Strike message, as evident in the minutes of a meeting held by members of the Lucky Strike Group at Lord & Thomas:

GRIFFIN: In all cases, we would like to get the plug for the thing a certain person intends to be plugged for, in the testimonial.

COONS: That’s a good point. But, it must be done in a clever way, and everything must be sincere and completely believable…

GRIFFIN: …I think the policy on the plugs should be that there will be put in a plug for their show or activity only if they require it or if their particular reference is of interest by itself in the testimonial.

COONS: In other words, we don’t want to put a plug in about a class “B” picture no one is ever going to see.^52^

Accordingly, Lucky Strike underwrote national advertising for more than one in five “A” class (big budget, top bill) pictures released in 1937 by the major studios, including 35 films from MGM, Paramount, RKO and Warner Bros (table 3).^61^ The movie tie-in and publication timing of Lucky Strike adverts were coordinated with the studios to deliver maximal promotional value. Proofs of Lucky Strike newspaper adverts are frequently dated a few days before a film’s opening in New York and other major cities.^62^ For example, in April 1937, Lord & Thomas informed movie star Gary Cooper that his magazine advert scheduled for “late June and early July” would “make mention of your Paramount Picture “Souls at Sea”, …Further we want to postpone your broadcast [on an American Tobacco-owned radio show] to a time shortly before the release of your Goldwyn picture “The Adventures of Marco Polo””.^63^

**Table 3:** US film studios engaged in tobacco cross-promotion, 1928–51

	Period	Extant adverts with studio plug
Major studios:		
Paramount	1931–1951	53
Warner Bros	1928–1950	34
Fox	1931–1951	29
MGM	1930–1951	28
RKO	1931–1937	21
Columbia	1931–1951	19
United Artists	1931–1949	18
Universal	1931–1951	13
Major studios total		215
Smaller studios		19
Hollywood testimonial adverts without explicit studio plugs		64

Smaller studios include David O Selznick, Enterprise, Eagle-Lion, First National, Pathé, Samuel Goldwyn, Santana and Radio Pictures. Total number of adverts containing studio plugs is a conservative estimate based on surviving records. An advertisement was counted if the studio name appeared in print or was mentioned on a tobacco company-sponsored radio program during the guest appearance of the Hollywood endorser.

Besides spending millions of dollars on advertising space and radio time to promote stars, their films and studios, American Tobacco paid Hollywood stars themselves at least US$218 750 (equivalent to US$3.2 million in 2008) in 1937 and 1938 to endorse Lucky Strikes in print adverts and on radio programs owned by American Tobacco (table 4). Top “A list” stars endorsing Lucky Strike were each paid US$73 000 (2008 equivalent) for their testimonial and benefited from national exposure—making them even more valuable to the studios and attractive to other national advertisers. American Tobacco often split payments into up front and year-end portions^63^ to ensure appearances by the stars on radio broadcasts, but several top stars negotiated a lump sum. Stars frequently extended their 1-year Lucky Strike agreements and presumably received a repeat payment to endorse Lucky Strike exclusively.^63^ In a standard agreement, American Tobacco supplied the actor with Lucky Strikes for a year,^63^ a modest gift for stars at this income level. Free cigarettes might have aided publicity or served as evidence that the star valued and smoked the brand, should these arrangements again be investigated.

**Table 4:** Lucky Strike’s paid Hollywood endorsements, 1937–8

Actor	Movie(s) and studio(s) promoted	Payment (US$)	2008 value (US$)
Beery, Wallace^70^	The Mad Man of Brimstone (MGM)	$10 000	$146 583
Bennett, Constance^63^	Topper (MGM)	$6000	$87 950
Boyer, Charles^71^	Tovarich (Warner Bros)	$3000	$43 975
Carroll, Madeleine^63^	The Prisoner of Zenda (Selznick)	$3000	$43 975
Claire, Marion^62^		$750	$10 994
Colbert, Claudette^63^	Maid of Salem (Paramount), I Met Him in Paris (Paramount)	$10 000	$146 583
Cooper, Gary^63^	The Adventures of Marco Polo (MGM), Souls At Sea (Paramount)	$10 000	$146 583
Crawford, Joan^63^	The Bride Wore Red (MGM)	$10 000	$146 583
Eilers, Sally^47^	We Have Our Moments (Universal)	$3000	$43 975
Fonda, Henry^72^		$3000	$43 975
Gable, Clark^69^	Saratoga (MGM)	$10 000	$146 583
Gaxton, William^62^		$1250	$18 323
Hope, Bob^72^		$2500	$36 646
Hopkins, Miriam 63	The Woman I Love (RKO)	$5000	$73 292
Lawrence, Gertrude^68^		$1750	$25 652
Lombard, Carole^63^	Swing High, Swing Low (Paramount), True Confession (Paramount)	$10 000	$146 583
Loy, Myrna^63^	Man Proof (MGM), Double Wedding (MGM)	$10 000	$146 583
MacMurray, Fred^59^	Exclusive (Paramount)	$6000	$87 950
Marshall, Herbert^63^	Angel (Paramount), A Love Like That (RKO)	$10 000	$146 583
McLaglen, Victor^59^	Cavalcade (20th Century Fox), Wee Willie Winkie (20th Century Fox)	$6000	$87 950
Merivale, Philip^59^		$3000	$43 975
Michael, Gertrude^59^		$2000	$29 317
Milland, Ray^59^		$2000	$29 317
Montgomery, Robert^59^	Live, Love, and Learn (MGM)	$10 000	$146 583
Nagel, Conrad^73^		$1500	$21 988
Navarro, Ramon^73^		$1500	$21 988
Powell, Richard^59^	Hollywood Hotel (Warner Bros)	$5000	$73 292
Raft, George^74^		$3000	$43 975
Raymond, Gene^74^	Three on A Latchkey (RKO)	$3000	$43 975
Rhodes, Erik^74^		$2000	$29 317
Robinson, Edward^74^	Kid Galahad (Warner Bros)	$3000	$43 975
Ross, Shirley^74^		$3000	$43 975
Ruggles, Charles^74^	Turn Off the Moon (aka Honeymoon Cottage) (Paramount)	$3000	$43 975
Sothern, Ann^75^	She’s Got Everything (RKO), Don’t Forget to Remember (RKO)	$3000	$43 975
Stanwyck, Barbara^76^	The Plough and the Stars (RKO)	$10 000	$146 583
Sullivan, Margaret^77^		$10 000	$146 583
Swanson, Gloria^75^		$1500	$21 988
Taylor, Robert^63^	Broadway Melody of 1938 (MGM), Yank at Oxford (MGM)	$10 000	$146 583
Tobin, Genevieve^78^		$3000	$43 975
Tracy, Spencer^78^	Captains Courageous (MGM), Mannequin (MGM)	$10 000	$146 583
Worth, Constance^79^		$2000	$29 317
Wyatt, Jane^79^	Lost Horizon (Columbia)	$6000	$87 950
Total		$218 750	$3 208 518

This list only includes actors who endorsed Lucky Strike in advertisements and for whom pay agreements exist today. For example, actors Cary Grant, Janet Gaynor and Bette Davis appeared in Lucky Strike adverts in 1937,^27^ but their endorsement contracts were not found.

The Lucky Strike campaign was not Hollywood’s only collaboration with tobacco advertisers. In 1937, American Tobacco bought US$58 000 worth of time (equivalent to US$872 546 in 2008) for seven in-theatre commercials that starred non-studio affiliated performers Genevieve Tobin and Buddy Rogers.^64^ ^65^ These commercials were primarily shown in independent theatres following a 1931 MPPDA decision to discourage advert films, which had received enough public backlash that the MPPDA feared further federal regulation.^51^ Before the MPPDA’s ruling, Paramount and Warner Bros had tested single-reel advertising films and planned to charge national advertisers a set price per 1000 viewers in the studio-owned theatre chains.^66^ The *New York Times* calculated that Liggett & Myers would have paid Paramount US$325 000 (equivalent to US$4.5 million in 2008) for a 13-film series advertising Liggett & Myers cigarettes.^66^

### Studios control deals with contract stars

While American Tobacco paid for national Hollywood campaigns, studio talent contracts gave studios complete control over the use of their celebrity “brand names”. Major studios negotiated the content of testimonials, insisted that the timing of adverts and radio appearances be coordinated with movie releases, and denied permission for deals that did not serve their interest. Paramount wrote to Lord & Thomas in September 1931, authorising “the use of a star’s name, likeness and testimonial” and stipulating that “all advertising, publicity, and exploitation matter [of Lucky Strike Cigarettes] mentioning the name or showing a likeness of [the actor] must first be submitted to this corporation for written approval before being used”.^67^ MGM, too, informed Lord & Thomas in July 1937, “It is important no advertisements are to be scheduled for publication until approval has been given by us, and until any changes we may wish to make concerning picture credit have been completed”.^68^ MGM also exercised power when it denied Clark Gable’s guest appearance on an American Tobacco radio show.^69^ Still, Lord & Thomas paid the balance promised to Gable and extended his Lucky Strike endorsement contract for another year.^69^

In July 1937, RKO permitted contract player Herbert Marshall to endorse Lucky Strike “upon the following conditions”:

(a) …[Y]ou will be announced as “Herbert Marshall, now co-starring with Barbara Stanwyck in RKO’s motion picture “A Love Like That” or if the name of the motion picture “A Love Like That” is subsequently changed, the changed title will be inserted;

(b) That in connection with magazine advertisements the said motion picture “A Love Like That” will also be announced and that the magazine advertisements will be released contemporaneously with the release of the said motion picture;

(c) That wherever possible, said motion picture will be announced in connection with the Lucky Strike Hit Parade radio hour.^63^

Correspondence detailing similar promotional specifications are preserved from Selznick International Pictures,^80^ Warner Bros,^80^^–^82 United Artists,^83^ The Samuel Goldwyn Company,^83^ 20th Century Fox Film^84^ and Paramount.^19^ ^85^

### Radio says Hollywood smokes Luckies, 1937

In the fall of 1937, coinciding with Lucky Strike’s Hollywood campaign, Lord & Thomas paid Warner Bros US$935 000^86^ (equivalent to US$13.7 million in 2008) to create *Your Hollywood Parade*, an hour-long weekly radio show for American Tobacco broadcasted from the Warner Bros lot. The program strung together acted out scenes from upcoming Warner Bros movies, according to the production agreement:

There shall be no previews of motion pictures other than Warner Bros pictures…Each such preview…shall as far as possible be presented by the stars or featured players featured in such Warner Bros picture…In addition, Warner Bros…agree to furnish such other members of their organization as may be selected by mutual consent to provide motion picture studio atmosphere, it being intended that the entire personnel of Warner Bros, except executives, shall be available for this purpose.^87^

For its part, Warner Bros declared, “Warner Bros believes that it will be to its advantage to cooperate in the broadcasting of such a program”, which was created at American Tobacco’s expense. Lord & Thomas supervised all aspects of the show and could cancel it if Warner Bros’ cooperation was unsatisfactory, or for any other reason.

American Tobacco’s radio programs were hard sell: in 1943, an American Tobacco radio producer catalogued 268 “Lucky Strike impressions” in 135 min of broadcast time, the equivalent of hearing the Lucky Strike brand name or jingle every 30 s.^88^ On *Your Hollywood Parade*, Warner Bros stars appeared, often delivering their testimonial, in Lucky Strike commercials delivered by emcee Dick Powell, a Warner Bros contract actor. The radio show reinforced the impression, also encouraged by the print campaign, that everyone in Hollywood smoked Lucky Strike—and that cigarettes seen onscreen were Luckies. For example:

I once asked a “property” man—who supplies cigarettes to the actors—what the favorite is. He answered by opening up a box containing cigarettes. In it were nothing but Luckies.—Testimonial signed by Miriam Hopkins, February 3, 1937.^63^

It’s always easy for me to get a Lucky from Joan Crawford or Clark Gable, or even from most of the newcomers to the studio…So, all in all, you can see I’m really enthusiastic.—Statement signed by Myrna Loy, December 28, 1937.^63^

In 1944, American Tobacco created *The Jack Benny Program*, contracting with the top-rated comic to deliver 105 30 min radio shows over 3 years for US$2.3 million (US$27.6 million in 2008). American Tobacco also deposited US$600 000 (US$7.2 million in 2008) into a “Special Exploitation Fund” to use as:

[T]he Contractor [Benny]…in his sole discretion may deem proper, including (but not limited to) for purposes of paying the compensation of guest artists who appear on the broadcasts *(the employment of guest artists from time to time being deemed desirable in connection with the exploitation of Sponsor [American Tobacco] and Sponsor’s products)*…it being expressly agreed that Contractor, in such advertising and exploitation, shall not be obligated to refer to or mention Sponsor or its products.^89^ (Emphasis added.)

**Figure 3:**
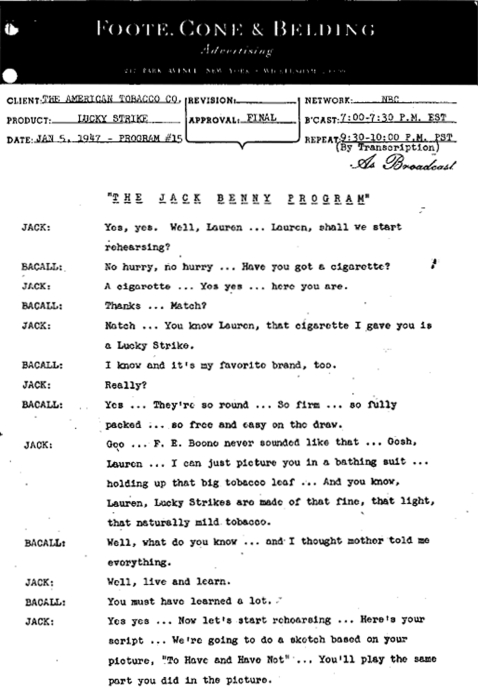
Perception of brand preference and use among Hollywood stars were supported by radio endorsements. American Tobacco’s Lucky Strike brand sponsored *The Jack Benny Program* from the mid-1940s to mid-1950s. This radio transcription from the January 5, 1947 broadcast is an example of a guest appearance and testimonial, given here by actress Lauren Bacall. Bacall mentions Lucky Strike is her favourite brand of cigarette. Stating her brand preference may have served to create an association between Lucky Strike and onscreen smoking by Bacall’s characters (not to mention in real life). The transcription also shows that Bacall’s guest appearance included a “sketch based on [her Warner Bros] picture “To Have and Have Not”” (1944), which co-starred Humphrey Bogart. Source: American Tobacco Company.^91^

The “Special Exploitation Fund” gave American Tobacco oversight and deniability for “guest star” Lucky Strike commercials. Channelling endorsement fees through the program’s producer may have temporarily avoided scrutiny by the FTC, which had launched another investigation into cigarette advertising in 1942.^90^ A sketch between Benny and Lauren Bacall on a January 1947 broadcast of *The Jack Benny Program*^91^ seamlessly promotes Lucky Strike and Bacall’s new film (fig 3).

### Chesterfield goes to Hollywood, 1946

When the FTC began investigating advertising methods of American, Lorillard and Reynolds in 1942 (Reynolds specifically for its Camel testimonial payments^92^^–^94), Liggett & Myers, makers of Chesterfield and the third largest cigarette company at the time, launched a multiyear Hollywood testimonial campaign in print and on radio, spending US$4.7 million (US$50.9 million in 2008) in 1946 alone.^95^ That year, Liggett spent more to advertise Hollywood than Paramount, 20th Century Fox, Warner Bros and Columbia Pictures—Liggett’s main Hollywood studio beneficiaries—combined.^95^

Chesterfield gained endorsements from Hollywood stars who formerly endorsed Lucky Strikes, including Barbara Stanwyck, Claudette Colbert, Gary Cooper, Bob Hope and Ray Milland at Paramount, Clark Gable at MGM, Fred MacMurray at Universal and Joan Crawford at Warner Bros. On the *Chesterfield Supper Club* radio program, many stars, such as Stanwyck and Susan Hayward, had their testimonials read by an unidentified actor. Others, such as Fred MacMurray and Rosalind Russell, delivered the commercial themselves. No payments are documented in Liggett’s files; presumably either the company or its advertising agency made arrangements directly with studios, or the payments to the stars were channelled through the radio show’s producers, as American Tobacco did at *The* *Jack Benny Program*.

## DISCUSSION

Smoking has appeared in movies since silent film,^10^ but the advent of “talking pictures” in the late 1920s marked the beginning of the American Tobacco Company’s systematic exploitation of film celebrities. Nearly 200 movie actors are known to have simultaneously promoted a tobacco brand and their studios’ releases from 1927–51; two-thirds of the top 50 box office stars in Hollywood from the late 1930s through the 1940s endorsed tobacco brands for advertising purposes.^96^ With these national testimonial advertisements, cigarette companies fostered the impression that cigarettes smoked by stars on screen were a specific brand. Tobacco companies, reported throughout this period to be targeting new women smokers to increase the size of the cigarette market,^97^ ^98^ used female film stars to model behaviour and increase social acceptance through testimonial advertising and onscreen smoking.^97^ ^99^

Major studios’ talent contracts^100^ ^101^ allowed them to maximise marketing opportunities by closely controlling their stars’ participation in some of the largest US advertising campaigns. Cross-promotion from cigarette advertising campaigns helped build studio brands, spotlight their biggest stars, and promote the big budget “A” class films at the top of theatre double bills. Tobacco campaigns also paid stars substantial sums while reinforcing the stars’ notoriety, boosting their value to the studios and other national advertisers. Free cigarettes provided under endorsement agreements created publicity opportunities on and off the set—a tobacco industry strategy revived in the 1980s.^9^ Despite the studios’ voluntary 1931 ban on product placement, the tobacco companies’ multimedia testimonial campaigns linked particular brands with actors, effectively branding “generic” cigarettes in films by advertising actors’ brand “preference”.

**What this paper adds**

- Smoking in movies is associated with adolescent and young adult smoking initiation.
- Public health efforts to reduce exposure to onscreen smoking are countered with arguments that tobacco imagery in “classic” movies was integral to filmmaking artistry.
- The present work explores the mutually beneficial commercial collaborations between the tobacco companies and major motion picture studios from the late 1920s to 1940s. We found endorsement contracts that reveal American Tobacco Company paid movie stars for their testimonials and negotiated cross-promotion with the studios to which the stars were contracted.
- The synergistic relationship between US tobacco and motion picture industries described in the present work grew out of cross-promotion incentives, and continues to perpetuate public tolerance of onscreen smoking.

The value of cigarette/movie tie-ins to the companies involved is difficult to monetise, but the fact that an estimated 20–25% of all major studios’ feature-length “A” class motion pictures appeared in Lucky Strike advertising in 1937 indicates the financial importance of these tie-ins to the studios.^14^ To participate in this lucrative partnership, the studios’ also repeatedly bypassed their own 1931 ban on actors’ product endorsements.^15^ In turn, American Tobacco Company and Liggett & Myers allocated portions of their multimillion US dollar budgets to print and radio campaigns featuring Hollywood stars, films and studios. This cultivated, synergistic relationship between Hollywood and the tobacco industry promoted social acceptance of smoking and, by explicitly and repeatedly associating Hollywood’s top stars with cigarette brands, made their motion pictures an integral part of the tobacco industry’s sales strategy.

By 1943, Reynolds, Liggett and American ranked among the nation’s top 10 advertisers overall. The 6 largest cigarette companies spent the 2008 equivalent of US$315 million to advertise that year, more than 10 times the US$28 million spent by the 8 major Hollywood studios.^13^

The tobacco and film industries’ mutual exploitation was not entirely unconstrained. Cigarette advertising provoked repeated federal inquiries into product claims and endorsement deals. Public criticism of product placement in films and commercials in theatres prompted self-regulatory policies from major studios. Tobacco companies adapted to increasing regulation and scrutiny by making legally prudential changes on paper, but continued to write and pay for endorsements.

Several factors may explain the decline of smoking frequency in US films after 1950 and until 1980,^102^ including publicity about diseases linked to smoking, the rapid penetration of advertising-driven television and the consequent shift of tobacco advertising and sponsorship dollars, and the breakdown of studio control over stars and theatre networks.

The legacy of cross-promotion during the “Golden Age” of Hollywood, led by American Tobacco and its advertising agency, Lord & Thomas, continues to be used to rationalise smoking as integral to the art of film making. Evidence suggests that this integration was a commercial collaboration “signed, sealed and delivered” (as Lucky Strike endorsement agreements from the 1930s put it) by the tobacco companies, major studios and many of the era’s best remembered stars. The failure of federal regulations and voluntary film industry policies to resist tobacco–film industry cross-promotion during the mid-20th century was followed by an increase in onscreen tobacco incidence after 1980, despite exposure of tobacco industry practices with the 1989 Congressional inquiry on product placement and nominal limitations in the 1998 Master Settlement Agreement.

Whereas legal and regulatory approaches, along with appeals to film “creatives” who lack control over film content and product placements, have failed to break the deliberately fostered association between Hollywood films and cigarettes since 1927, current broad-based efforts to create market disincentives within the film industry, specifically by rating future smoking “R”, could prove more effective.

The presumption promoted by those who oppose rating future smoking “R” is that mainstream motion pictures are an art form into which social agendas should not intrude. The pattern of close cooperation between the film and tobacco industries, from the advent of sound in 1927 to the transfer of tobacco sponsorship to television starting in the late 1940s and the re-emergence of film–tobacco deals after tobacco adverts were barred from television in the 1970s, suggests instead that the motion picture industry was always ready to cater to the tobacco industry’s commercial agenda.

As in the 1930s, nothing today prevents the global tobacco industry from influencing the film industry in any number of ways to achieve its own strategic objectives. It would be more accurate to view motion pictures (and video programming) not as disinterested artistic works but as commercial platforms (which occasionally achieve the status of art) serving a variety of agendas, not all of which — as in the case of product placement deals struck by producers — consistently respect the work’s artistic integrity or the unsuspecting audience in search of entertainment or inspiration. Policy makers who recognise the historic and contemporary role played by Hollywood films in expanding and renewing the market for tobacco products should not hesitate to modernise rating systems to exclude smoking from films marketed to youth, thereby taking steps necessary to break the long standing commercial connection between movies and smoking.
